# Nomograms for predicting progression and efficacy of post-operation radiotherapy in IIIA-pN2 non-small cell lung cancer patients

**DOI:** 10.18632/oncotarget.16564

**Published:** 2017-03-25

**Authors:** Baozhong Zhang, Zhiyong Yuan, Lujun Zhao, Qingsong Pang, Ping Wang

**Affiliations:** ^1^ Department of Radiotherapy, Tianjin Medical University Cancer Institute and Hospital, National Clinical Research Center for Cancer, Tianjin Key Laboratory of Cancer Prevention and Therapy, Tianjin's Clinical Research Center for Cancer, Tianjin, People's Republic of China

**Keywords:** non-small cell lung cancer (NSCLC), N2, post-operative chemo-radiotherapy (POCRT), nomogram

## Abstract

In this retrospective study, we developed nomograms for predicting the efficacy of post-operation radiotherapy (PORT) in IIIA-N2 non-small cell lung cancer (NSCLC) patients. In total, 334 patients received post-operational chemotherapy and were included in the analysis. Of those, 115 also received either concurrent or sequential post-operational radiotherapy (PORT). Nomograms were developed using Cox proportional hazard regression models to identify clinicopathological characteristics that predicted progression free survival (PFS) and overall survival (OS), and subgroup analyses of the effects of PORT were performed using nomogram risk scores. PFS and OS predicted using the nomogram agreed well with actual PFS and OS, and patients with high PFS/OS nomogram scores had poorer prognoses. In subgroup analyses, PORT increased survival more in patients with low PFS nomogram risk scores or high OS nomogram risk scores. Thus, our novel nomogram risk score model predicted PFS, OS, and the efficacy of PORT in IIIA-N2 NSCLC patients.

## INTRODUCTION

Patients with stage IIIA pN2 non-small cell lung cancer (NSCLC) differ in clinicopathologic characteristics and in the risk of local recurrence and metastasis after complete resections. Although the efficacy of post-operative chemotherapy (POCT) after complete resection has been confirmed in stage II and III NSCLC patients [3–5], the value of post-operative radiotherapy (PORT) in these patients remains controversial [1, 2]. An early meta-analysis found that PORT was detrimental to patients with completely resected NSCLC, especially for those with stage I/II N0-N1 disease [6]; PORT therefore declined in popularity as a treatment for NSCLC patients for several years. But in 2006, Lally *et al*. [7] demonstrated that PORT was beneficial for pN2 NSCLC patients. The Adjuvant Navelbine International Trialist Association (ANITA) trial confirmed that PORT was associated with better overall survival (OS) in patients with resected pN2 NSCLC [8]. Overall survival of IIIA-pN2 NSCLC patients is generally poor; the 5-year OS rate for such patients in the SEER database is 24% [9]. However, additional studies are needed to confirm the efficacy of PORT for treating IIIA-pN2 NSCLC patients. A robust prognostic model for predicting prognosis would help determine the efficacy of PORT in IIIA-N2 NSCLC patients. In this study, we developed a nomogram based on clinical features for predicting prognosis and the value of PORT for treating stage IIIA-N2 NSCLC patients.

## RESULTS

Clinicopathological characteristics for the 334 patients included in the survival analysis after filtering are shown in Table 1. The 115 patients who received POCRT were assigned to the PORT group, while the 219 patients received only POCT were assigned to the non-PORT group. The median PFS and OS for all 334 patients were 16.0 months (95% CI: 13.982–18.018) and 36.0 months (95% CI: 30.615–41.385), respectively; the 3-year PFS and OS probability were 25.7% and 48.7%, respectively.

**Table 1:** Clinicopathological characteristics

Variable		**PORT**	Non-PORT
Gender	Male	83	136
	Female	32	83
Age	≤ 60	75	125
	> 60	40	94
Pathology	Adenocarcinoma	54	131
	Squamous carcinoma	46	58
	Adeno-squamous carcinoma	5	18
	others	10	12
Surgical procedure	Pneumonectomy	13	29
	Lobectomy/Tumor excision	102	190
T stage	T1	40	72
	T2	52	121
	T3	23	26
Number of positive N2 stations	Single	54	117
	Multiple	61	102
Extra capsular extension	Positive	30	37
	Negative	85	182
Positive total lymph nodes’ ratio	≤ 25%	63	121
	> 25%	52	98
Positive N2 lymph nodes’ ratio	≤ 20.5%	55	110
	> 20.5%	60	109
Lymph node skip	No skip	62	141
	Skip	53	78
Dissected N2 stations	≥ 6	79	166
	≥ 6	36	53
Total		115	219

### PORT is associated with increases in survival

**Figure 1:**
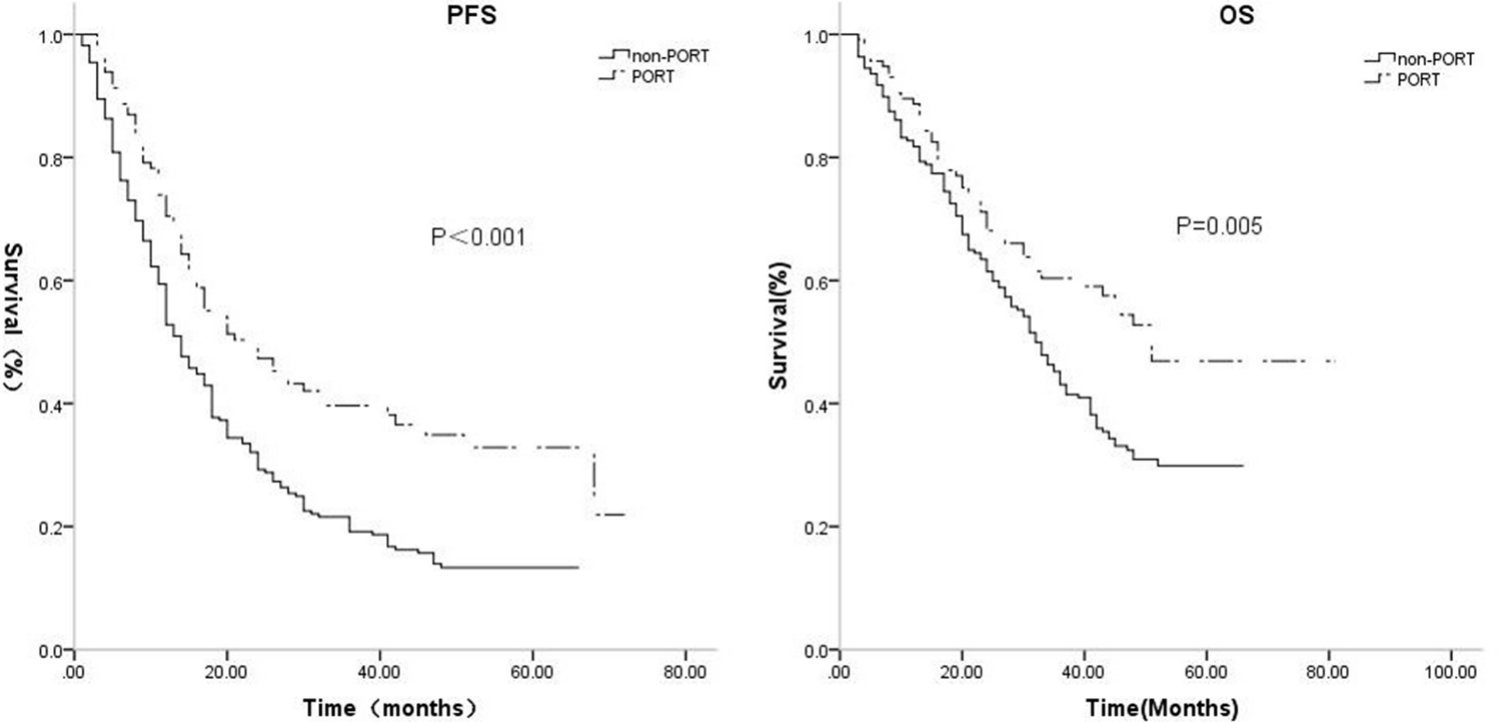
The median, 3-year PFS were respectively, 24 months, 39.7% for the PORT group, while 14 months, 19.2% for the non-PORT group (*p* = 0.000) The median, 3-year OS were respectively, 51 months, 60.4% for the PORT group, while 32 months, 43.1% for the non-PORT group (*p* = 0.005).

PORT group patients, with median 3-year PFS duration and probability of 24 months and 39.7%, had improved survival compared to the non-PORT group, with a median 3-year PFS duration and probability 14 months and 19.2% (*p* < 0.001). Median 3-year OS was also better in PORT patients (duration: 51 months, rate: 60.4%) than in non-PORT patients (duration: 32 months, rate: 43.1%, *p* = 0.005, Table 2, Figure 1).

**Table 2:** Survival based on PORT

		PORT	Non-PORT	
PFS	Median	24	14	*P* = 0.000
	3-year PFS	39.7%	19.2%	
OS	Median	51	32	*P* = 0.005
	3-year OS	60.4%	41.3%	

### Nomograms for predicting PFS and OS

**Figure 2:**
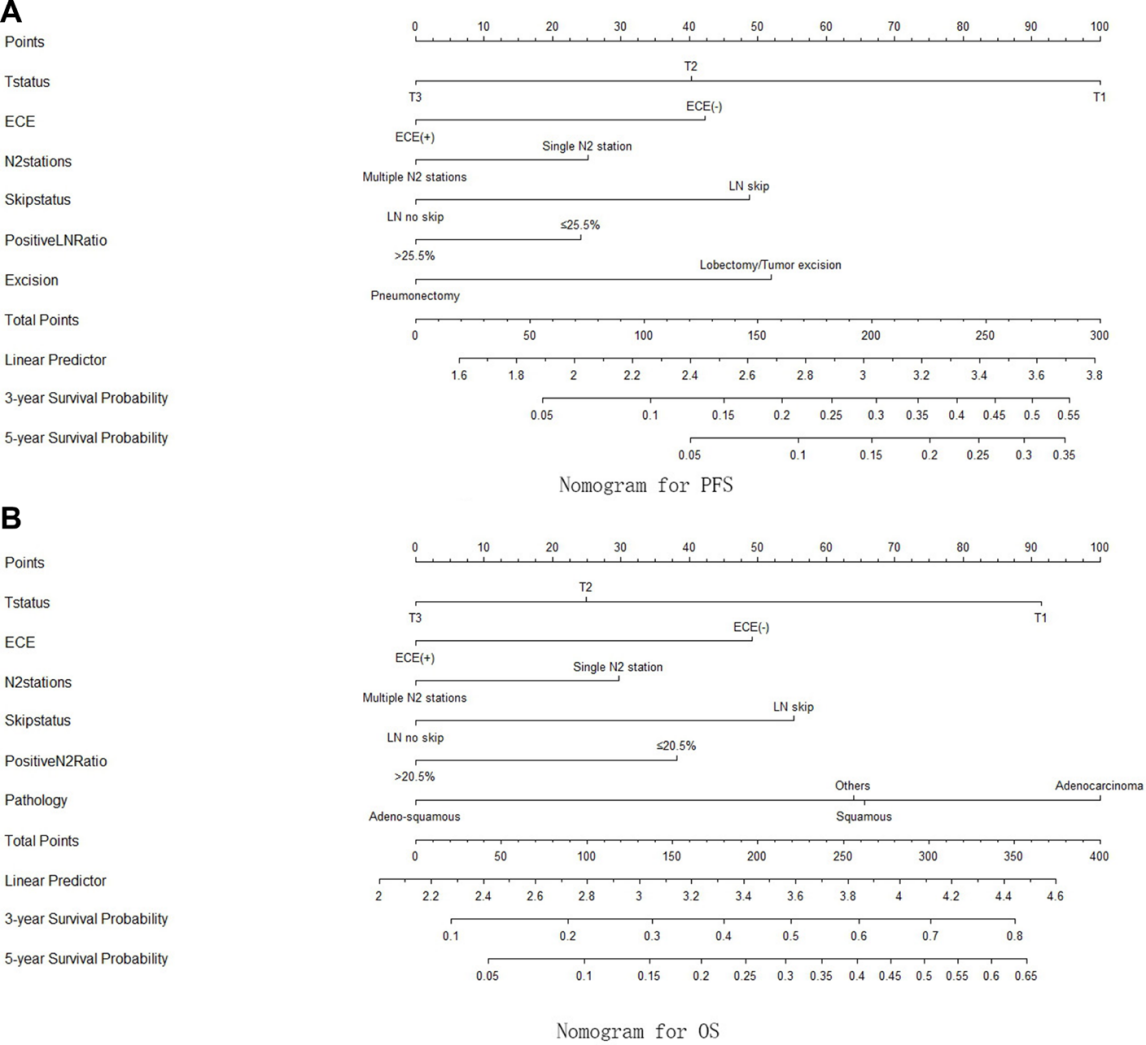
(**A**) The nomogram for PFS, constructed based on T stage, ECE, posivive N2 station number, LN skip status, positive LN ratio and the surgical procedures; (**B**) the nomogram for OS, constructed based on T stage, ECE, posivive N2 station number, LN skip status, positive N2 ratio and pathology.

**Figure 3:**
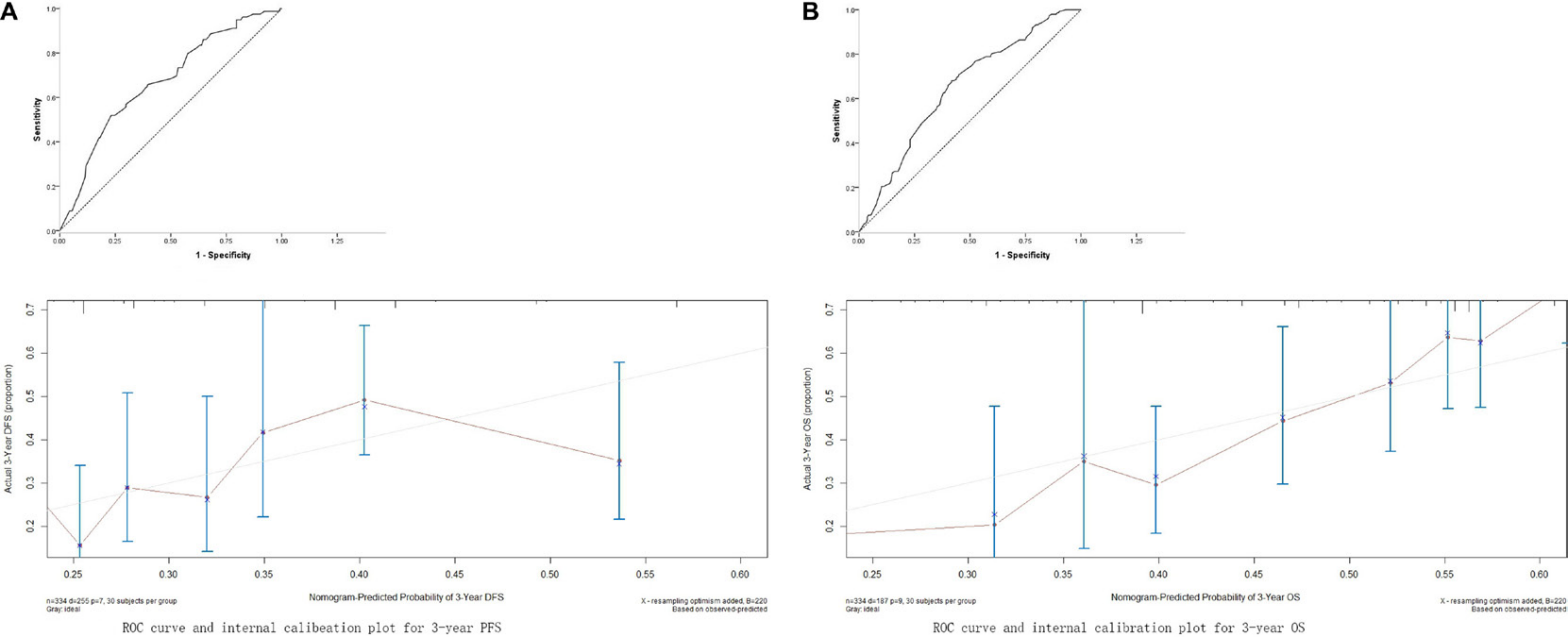
Receiver operating characteristic (ROC) curve and internal calibration plot (**A**) for the 3-year PFS, area under Receiver operating characteristic (ROC) curve was 0.670 (95% CI: 0.603–0.737), internal calibration plot C-index = 0.653, *p* = 0.037; (**B**) for the 3-year OS, area under ROC curve was 0.647 (95% CI: 0.589–0.706), C-index = 0.649, *p* = 0.044.

The nomograms for predicting PFS and OS were constructed using the Cox model. The results of univariate and multivariate Cox analyses are shown in Table 3A and 3B. The nomogram for predicting PFS was constructed based on T stage, extra-capsular extension (ECE), positive N2 station number, lymph node (LN) skip status, positive LN ratio, and surgical procedure, while the nomogram for predicting OS was constructed based on T stage, ECE, positive N2 station number, LN skip status, positive N2 ratio, and pathology (Figure 2). The nomograms were used to predict for 3-year and 5-year PFS and OS probabilities; associated ROC curves and internal calibration plots are shown in Figure 3. The area under the multivariate model ROC curve for PFS was 0.670 (95% CI: 0.603–0.737), and the calibration plot showed good agreement between predicted and observed 3-year PFS (C-index = 0.653, *p* = 0.037). The area under the multivariate model ROC curve for OS was 0.647 (95% CI: 0.589–0.706), and the calibration plot also showed good agreement between predicted and observed 3-year OS (C-index = 0.649, *p* = 0.044).

**Table 3A:** Univariate and multivariate Cox proportional hazard regression analysis-between clinicopathologic variables and PFS

Variables		Univariate			Multivariate		
		HR	95% CI	*P* value	HR	95% CI	*P* value
T status	T1	1			1		
	T2	1.606	1.212–2.127	0.001	1.610	1.206–2.147	0.001
	T3	2.345	1.585–3.469	0.000	2.076	1.339–3.219	0.001
ECE	ECE (−)	1			1		
	ECE (+)	1.602	1.184–2.167	0.002	1.372	0.978–1.926	0.067
N2 stations	Single	1			1		
	Multiple	1.372	1.073–1.756	0.012	1.162	0.890–1.518	0.270
Excision	Lobectomy/Tumor resction	1			1		
	Pneumonectomy	1.729	1.213–2.465	0.002	1.168	1.109–2.330	0.012
LN Skip	LN skip	1			1		
status	LN no skip	1.539	1.186–1.997	0.001	1.618	1.216–2.152	0.001
Positive	≤ 25.5%	1			1		
LN ratio	> 25.5%	1.485	1.160–1.900	0.002	1.232	0.928–1.636	0.148

**Table 3B:** Univariate and multivariate Cox proportional hazard regression analysis-between clinicopathologic variables and OS

Variables		Univariate			Multivariate		
		HR	95% CI	*P* value	HR	95% CI	*P* value
T status	T1	1			1		
	T2	1.613	1.160–2.242	0.005	1.700	1.207–2.395	0.002
	T3	2.142	1.341–3.422	0.001	2.059	1.232–3.441	0.006
ECE	ECE (−)	1			1		
	ECE(+)	1.574	1.108–2.236	0.011	1.377	0.942–2.015	0.099
N2 stations	Single	1			1		
	Multiple	1.542	1.155–2.058	0.003	1.217	0.874–1.695	0.245
Pathology	Adenocarcinoma	1			1		
	Squamous carcinoma	1.176	0.852–1.624	0.324	1.355	0.966–1.899	0.078
	Adeno-squamous carcinoma	1.996	1.219–3.269	0.006	2.322	1.404–3.839	0.001
	others	1.251	0.700–2.234	0.450	1.353	0.754–2.429	0.311
LN Skip	LN skip	1			1		
status	LN no skip	1.486	1.095–2.018	0.011	1.592	1.154–2.197	0.005
Positive	≤ 20.5%	1			1		
N2 ratio	> 20.5%	1.515	1.134–2.023	0.005	1.491	1.052–2.113	0.025

### Nomogram scores predict the efficacy of PORT in subgroup analysis

Based on ROC analysis of the nomogram, a cutoff value of 215 was selected for predicting PFS, and patients were divided into low (group 1, 0–215) and high (group 2, > 215) PFS groups based on this cutoff. There were 234 patients in the low PFS group and 100 patients in the high PFS group. K-M analysis revealed that median PFS and 3-year PFS probability were higher in low PFS patients (12 months, 95%CI: 10.475–13.525, 17.8%, respectively) than in high PFS patients (30 months, 95%CI: 21.931–38.069, 43.8%, respectively, *p* = 0.000, Figure 5). The cutoff value for OS was 221; based on this cutoff, 156 patients were assigned to the low OS group (group 1, 0–221) and 178 patients were assigned to the high OS group (group 2, > 221). K-M analysis revealed that median OS and 3-year OS probability were also higher in low OS group patients (25 months, 95%CI: 20.352–29.648, 28.4%, respectively) than in high OS group patients (51 months, 95% CI: 45.615–59.382, 65.5%, respectively, *p* = 0.000).

**Figure 4:**
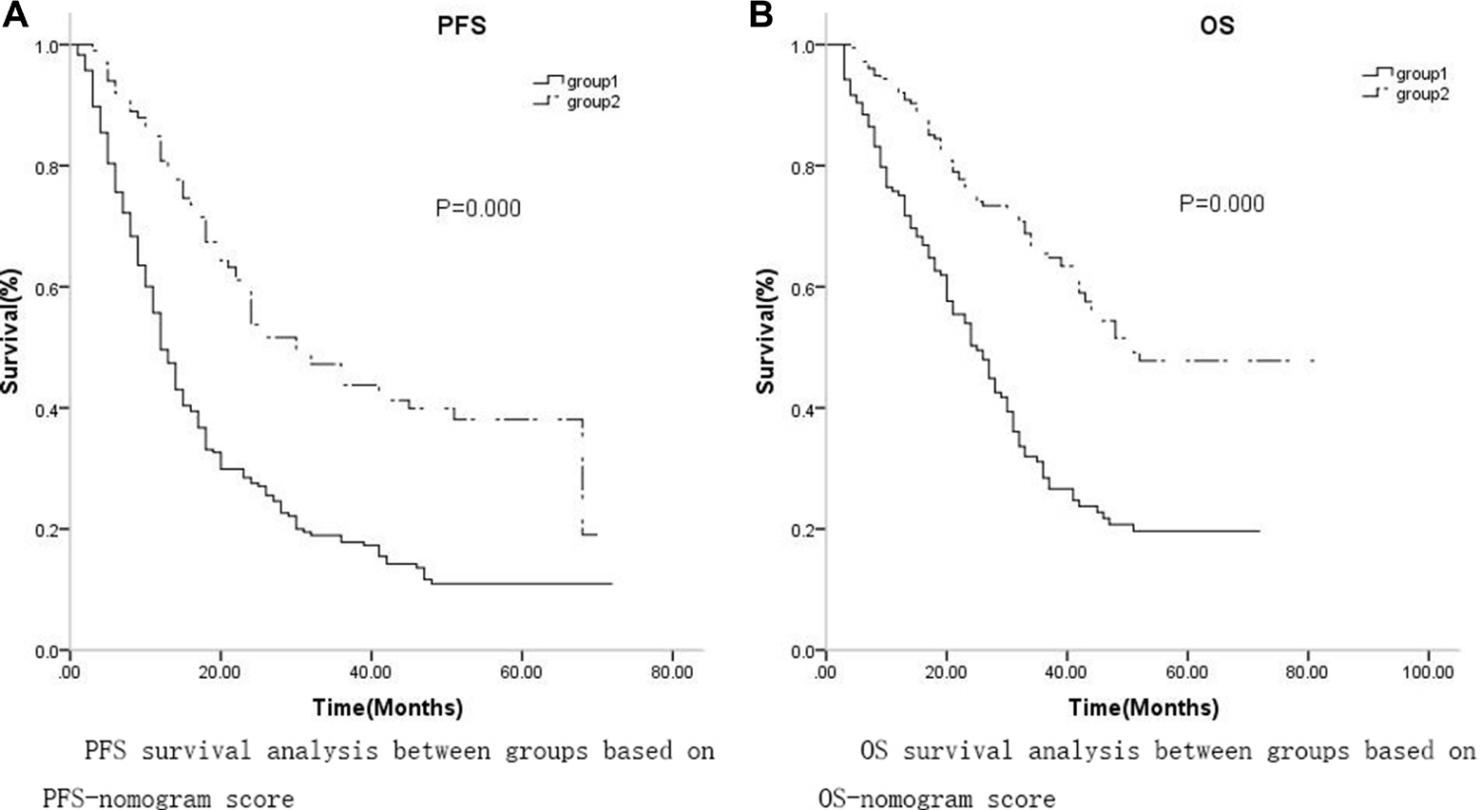
(**A**) The median PFS and 3-year PFS probability were lower in group 1 (12 months, 95% CI: 10.475–13.525, 17.8%, respectively) than in group 2 (30 months, 95% CI: 21.931–38.069, 43.8%, respectively, *p* = 0.000); (**B**)The median OS and 3-year OS probability were also lower in group 1 (25 months, 95% CI: 20.352–29.648, 28.4%, respectively) than in group 2 (51 months, 95% CI: 45.615–59.382, 65.5%, respectively, *p* = 0.000).

Subgroup K-M analysis was conducted to determine whether PORT improved survival. Among the low PFS patients, 80 received PORT and 154 did not. Low PFS patients who did not receive PORT had lower median PFS and 3-year PFS probability (12 months, 95% CI: 10.462–13.538, 11.7%, respectively) than low PFS patients who received PORT (17 months, 95% CI: 13.340–20.660, 31.5%, respectively, *p* = 0.000). Of the high PFS patients, 35 received PORT and 65 did not. High PFS patients who did not receive PORT also had lower median PFS and 3-year PFS probability (24 months, 95% CI: 19.558–28.412, 36.7%, respectively) than high PFS patients who received PORT (46 months, 95% CI: 26.660–58.962, 57.9%, respectively, *p* = 0.050, Table 4, Figure 4). Among the low OS patients, 51 received PORT and 105 did not. Median OS and 3-year OS probability tended to be lower in low OS patients who did not receive PORT (24 months, 95%CI: 18.830–29.170, 23.4%, respectively) than in low OS patients who received PORT (27 months, 95% CI: 21.017–32.983, 35.8%, respectively, *p* = 0.063), but this difference was not statistically significant. Among the high OS patients, 64 received PORT and 114 did not. High OS patients who did not receive PORT had lower median OS and 3-year OS probability (44 months, 95% CI: 37.909–50.091, 59.9%, respectively) than high OS patients who received PORT (56 months, 95% CI: 50.134–64.962, 76.8%, respectively, *p* = 0.029, Table 4, Figure 4).

**Table 4A:** Nomogram score-subgroup analysis of PFS for PORT

	Group 1		Group 2	
	PFS median (months)(95% CI)	3–year PFS probability	PFS median (months) (95% CI)	3-year PFS probability
Non-PORT	12 (10.462–13.538)	11.7%	24 (19.558–28.412)	36.7%
PORT	17 (13.340–20.660)	31.5%	46 (26.660–58.962)	57.9%
*P* value	0.000		0.050	

**Figure 5:**
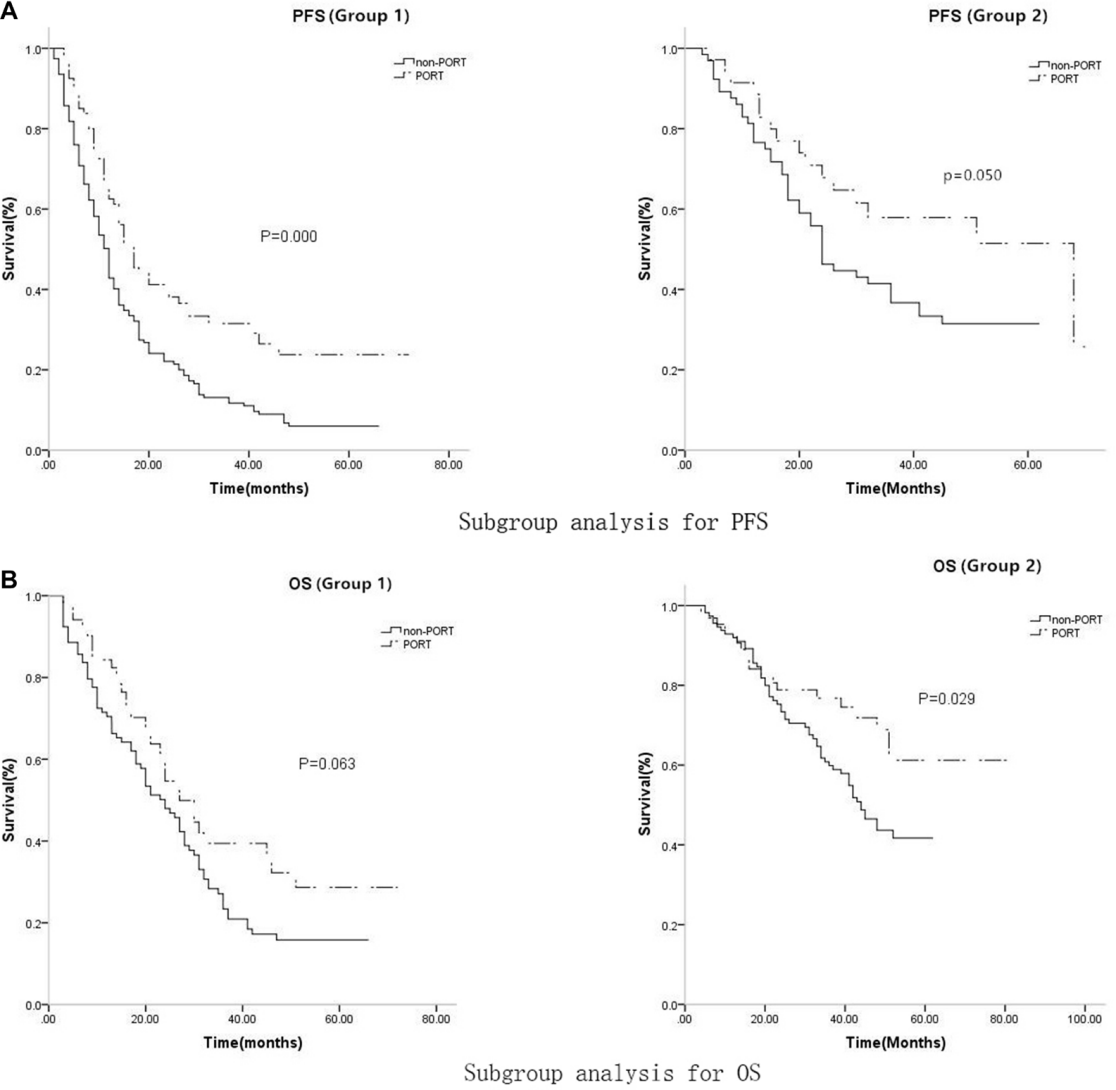
For PFS, In low-score group, the median PFS and 3-year PFS probability were 12 months (95%CI: 10.462-13.538) and 11.7% for non-PORT subgroup, while 17 months (95%CI: 13.340–20.660) and 31.5% for PORT subgroup respectively, (*p* = 0.000) In high-score group, the median PFS and 3-year PFS probability were 24 months(95% CI: 19.558–28.412) and 36.7% for non-PORT subgroup, while 46 months (95% CI: 26.660–58.962) and 57.9% for PORT subgroup respectively, (*p* = 0.050); for OS, In low-score group, the median OS and 3-year OS probability were 24 months (95% CI: 18.830–29.170) and 23.4% for non-PORT subgroup, while 27 months (95% CI: 21.017–32.983) and 35.8% for PORT subgroup respectively, (*p* = 0.063); In high-score group, the median OS and 3-year OS probability were 44 months (95%CI: 37.909–50.091) and 59.9%% for non-PORT subgroup, while 56 months (95% CI: 50.134–64.962) and 76.8% for PORT subgroup respectively, (*p* = 0.029).

**Table 4B:** Nomogram score-subgroup analysis of OS for PORT

	Group1		Group2	
	OS median (months)(95% CI)	3–year OS probability	OS median (months)(95% CI)	3-year OS probability
Non-PORT	24 (18.830–29.170)	23.4%	44 (37.909–50.091)	59.9%
PORT	27 (21.017–32.983)	35.8%	56 (50.134–64.962)	76.8%
*P* value	0.063		0.029	

## DISCUSSION

The efficacy of PORT after complete resection in stage IIIA pN2 NSCLC patients remains controversial. Some retrospective studies report that PORT improved local recurrence free survival (LRFS) or progression free survival (PFS), but had no impact on OS [10–12]. In contrast, C. Billiet *et al*. [13] reported that PORT improved OS in stage IIIA-N2 NSCLC patients. However, because they are a heterogeneous group with different clinicopathological features, patients with pN2 IIIA NSCLC would likely benefit from individualized treatments. Several clinical and pathological factors, including the number of pathologically involved lymph node (LN) stations [14, 15], positive lymph node ratio (LNR) [16–20], extra-capsular extension (ECE) [21], and LN skip status [22], are predictive of prognosis in NSCLC patients and should be considered when weighing the risks and benefits of PORT, which induces damage. It is particularly important to examine multiple factors when predicting patient outcomes and the therapeutic efficacy of PORT; no single factor is sufficiently accurate for such evaluations.

In this retrospective study, we identified risk factors for tumor recurrence after surgery in IIIA-N2 NSCLC patients and established a novel nomogram prediction model to estimate PFS probabilities and the efficacy of PORT. Similar nomogram prediction models have been used for several types of malignant tumors, including breast cancer, early stage non-small cell lung cancer, and osteosarcoma [23–27]. Here, K-M analysis revealed that patients who received PORT had better PFS and OS than patients who did not receive PORT. We then developed nomograms for predicting PFS and OS in R0 resected IIIA-N2 NSCLC patients. These predictive and prognostic models were internally validated, and calibration and discrimination tests indicated that they performed well. These nomograms may therefore be useful for risk assessments and for selecting individualized therapies. Subgroup analysis revealed that PORT increased median PFS by 5 months and increased 3-year PFS probability by 19.8% in the low PFS nomogram score group. Similarly, PORT increased median PFS by 22 months and increased 3-year PFS probability by 21.2% in the high PFS nomogram group. Furthermore, the smaller *p* value associated with the difference in the low PFS group compared to the high PFS group emphasizes the potential benefit of PORT for low PFS group patients in particular. In the high OS nomogram group, PORT increased median survival by 12 months and increased 3-year survival probability by 16.9%; there was a trend towards similar, albeit smaller, increases in the low OS nomogram group, but they did not reach statistical significance. Therefore, patients with high OS nomogram scores may benefit more from PORT.

This novel nomogram risk score system might assist in the selection of individualized treatments for IIIA-N2 NSCLC patients while avoiding unnecessary adverse effects. However, some limitations of this study should be considered when interpreting the results. First, although the nomogram was validated internally, it was not validated externally in an independent set of patients. Future studies should be conducted to determine whether it is applicable in other patient sets and populations [28]. Second, because this is a retrospective study, patients differed in the primary treatment they received, and these treatments might have affected the survival assessment. Randomized prospective studies with larger numbers of patients are crucial for confirming the utility of this nomogram risk score model for predicting PFS, OS, and the efficacy of PORT treatment in IIIA-N2 NSCLC patients.

## MATERIALS AND METHODS

Of the 388 IIIA-N2 NSCLC patients who underwent resection at Tianjin Medical Collage Cancer Hospital between Jan 1st, 2008 and Dec 30th, 2011, 334 were included in this retrospective analysis. Staging and pathologic identification were performed according to the 7th edition tumor node metastasis (TMN) staging system recommended by the IASLC (International Association for the Study of Lung Cancer) and the UICC (Union for International Cancer Control). All patients underwent CT or PET-CT scans before surgery to confirm either that they had lower than N2 status or, if they had N2 disease, that it was resectable. Only patients with N2 disease identified by postoperative pathology were included in the analysis. All patients received post-operative chemotherapy (POCT); 115 received postoperative chemoradiotherapy (POCRT, concurrent for 25 patients and sequential for 90 patients), while the remaining 219 received POCT only. The exclusion criteria included: disease status that exceed IIIA or pN2; multiple primary cancer; pretreatment before operation (chemotherapy and/or radiotherapy); incomplete resection (positive margin, surgical margin < 1.5 centimeter, or lymph node dissection < 3 stations); serious medical complications at the time of surgery; or a follow-up period of less than 3 months. Patient follow-ups occurred once every three months for the first two years following surgery and every six months thereafter during hospitalizations and/or outpatient clinic consultations. CT scans of the chest and MRIs of the head were performed at each follow-up, and local recurrence or distant metastasis was confirmed by pathology or PET if necessary. The chemotherapy regimens consisted of 4 cycles of intravenous docetaxel (75 mg/m^2^) or paclitaxel (175 mg/m^2^) and cisplatin (75 mg/m^2^) for non-adenocarcinoma patients and docetaxel (75 mg/m^2^), paclitaxel (175 mg/m^2^), or pemetrexed (500 mg/m^2^) and cisplatin (75 mg/m^2^) for adenocarcinoma patients, with an interval of 3 weeks. Radiation therapy consisted of 1.8 Gy per fraction for 28 fractions for a total dose of 50.4 Gy using linear accelerator, 6 MV X-rays. This study was approved by the Regional Ethics Committee of Tianjin Medical University Cancer Institute and Hospital, and all patients were contacted by telephone to obtain verbal informed consent.

### Statistical analysis

PFS was defined as the interval between the date of the initial operation and the date of progression or the last visit. OS was defined as the interval between the date of the initial operation and the date of death or the last visit. Variables correlated with disease progression were identified using univariate and multivariate COX analyses; variables with univariate *p* < 0.05 were included in the nomograms. The nomograms were built based on the Cox proportional hazards regression model for survival data. Receiver operating characteristic (ROC) curves and internal calibration plots were used to verify the nomogram score. Survival analysis was conducted using the Kaplan–Meier method, and *p* < 0.05 was considered statistically significant. Kaplan–Meier and the COX analysis were conducted with SPSS 17.0 software; nomogram, ROC, and internal validation were conducted with R software, version 3.2.2.

### Ethics approval

This study was approved by the Regional Ethics Committee of Tianjin Medical University Cancer Institute and Hospital (Reference number: TMC-RTR-110050), and all patients were contacted by telephone to obtain verbal informed consent.

### Availability of data and materials

The datasets generated for this study are available in the ‘MRAN’ platform (http://mran.microsoft.com), a complete open source platform for statistical analysis and data science, including the ‘R’ software and all packages.
